# Supplementary material for: Identification of New QTLs for Dietary Fiber Content in Aegilops biuncialis
Source: Int J Mol Sci. 2022 Mar 30;23(7):3821. doi: 10.3390/ijms23073821 (PMC8999039; doi:10.3390/ijms23073821)
Supplement: Supplementary file 1 [file ijms-23-03821-s001.zip › Supplementary-Materials.pdf]

# Identification of new QTLs for dietary fibre content in an *Aegilops biuncialis* collection through a GWAS approach

***Supplementary Material***

László Ivanizs[1†], Ilaria Marcotuli[2†], Marianna Rakszegi[1†], Balázs Kalapos[1], Kitti Szőke-Pázsi[1], András Farkas[1], Edina Türkösi[1], Eszter Gaál[1], Klaudia Kruppa[1], Péter Kovács[1], Éva Darkó[1], Éva Szakács[1*], Mahmoud Said[3], Jaroslav Doležel[3], Agata Gadaleta[2], István Molnár[1,3]

[1]Department of Biological Resources, Centre for Agricultural Research, Eötvös Loránd Research Network, Martonvásár 2462, Hungary

[2]Department of Agricultural and Environmental Science, University of Bari 'Aldo Moro', Via G. Amendola 165/A, 70126, Bari, Italy

[3]Institute of Experimental Botany of the Czech Acadey of Sciences, Centre of the Region Haná for Biotechnological and Agricultural Research, Olomouc, Czech Republic

[†]These authors are equal first author

**Table S1.**

Phenotypic data of grain quality traits (β-glucan, total and water-extractable pentosans, and protein content) measured in a collection of 83 *Aegilops biuncialis* accessions and the Mv9kr1 wheat genotype for two years.

**Table S2.**

Results of the BLASTn search of SNP-DArT markers associated with grain quality traits against the draft sequence assemblies of M and U genome chromosomes of *Ae. comosa* and *Ae. umbellulata*, respectively, and the reference sequences of hexaploid wheat chromosomes (Ensembl Plants, release-46).

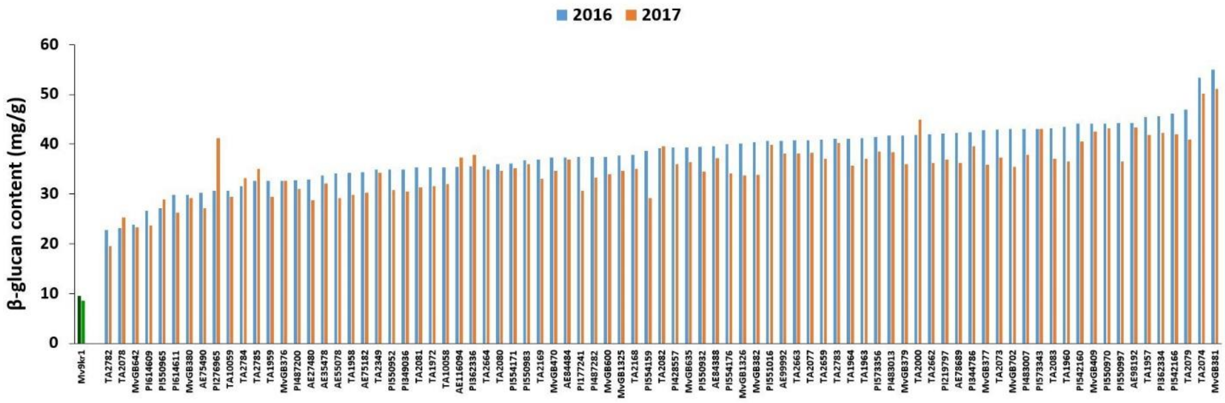


**Figure S1.** Intraspecific variation of β-glucan content in the *Ae. biuncialis* collection (2016, 2017, n=83). The β-glucan content of the Mv9kr1 wheat genotype was labeled with green (2016: dark-green, 2017: pale-green).

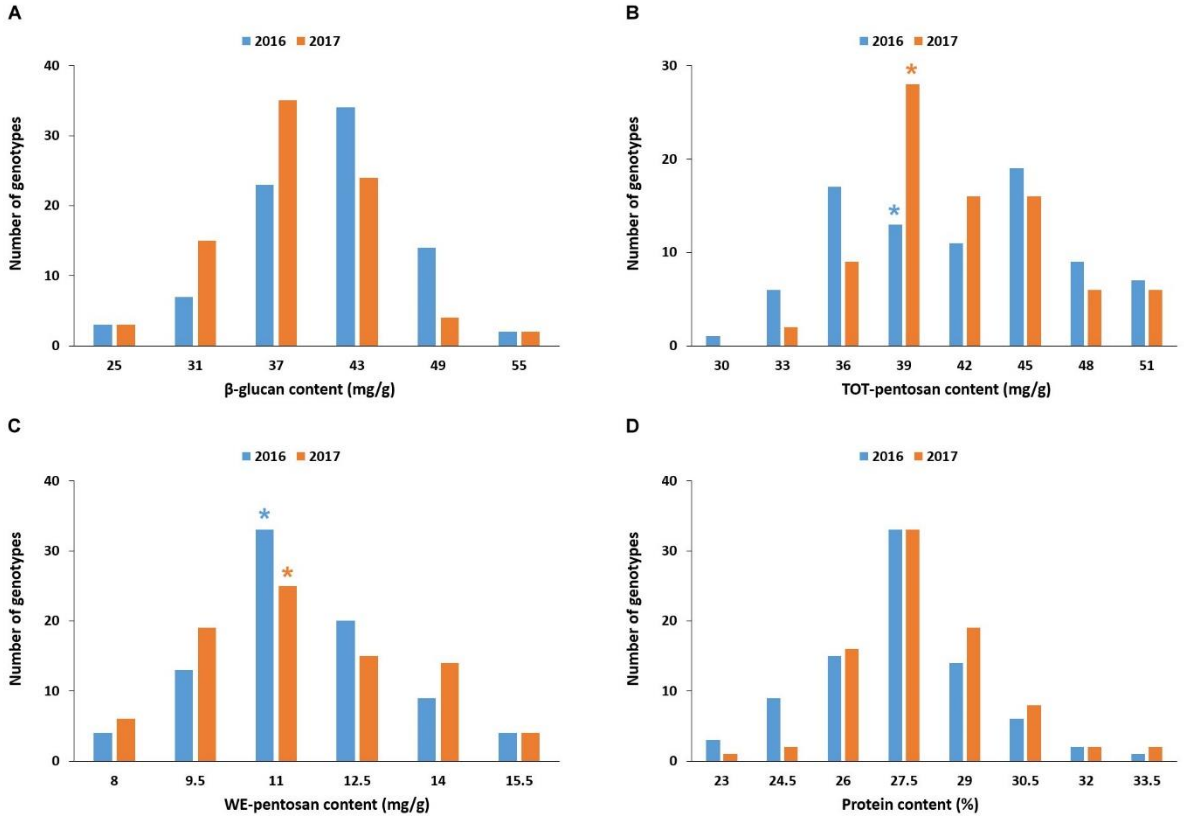


**Figure S2.** Frequency distribution of grain quality parameters in the *Ae. biuncialis* collection grown in Martonvásár (2016, 2017). For pentosan fractions, the location of the Mv9kr1 control wheat line has been indicated by stars on the relevant plots. The location of Mv9kr1 was not labeled for β-glucan and protein because of their extremely low values (β-glucan: 9.44 and 8.62 mg/g, protein: 12.91 and 13.01%).

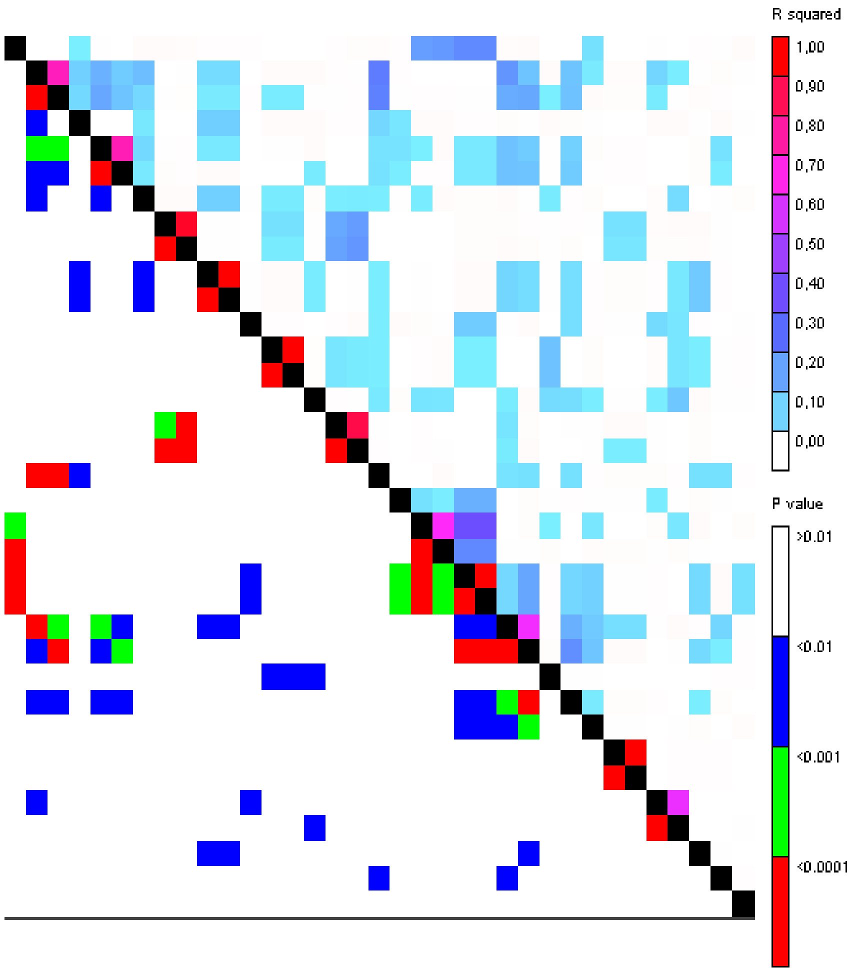


**Figure S3.** Linkage disequilibrium revealed by DArTSeq derived SNP markers in *Ae. biuncialis* population.

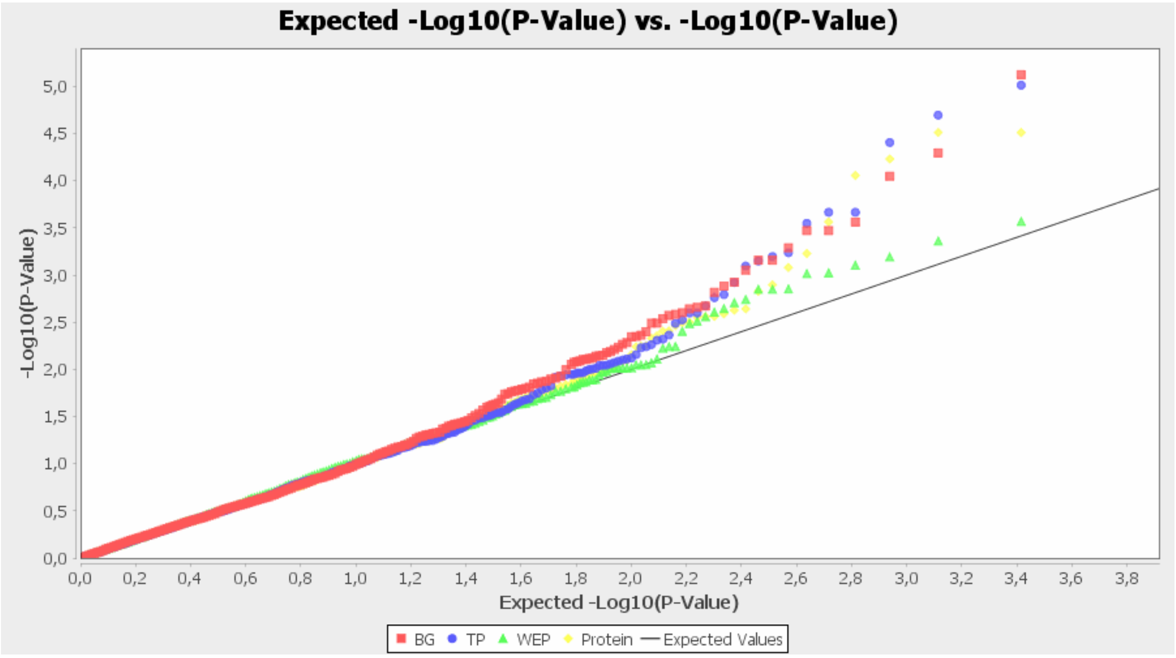

**Figure S4.** Q-Q plots generated for the four traits analyzed using the 83 *Ae. biuncialis* accessions.

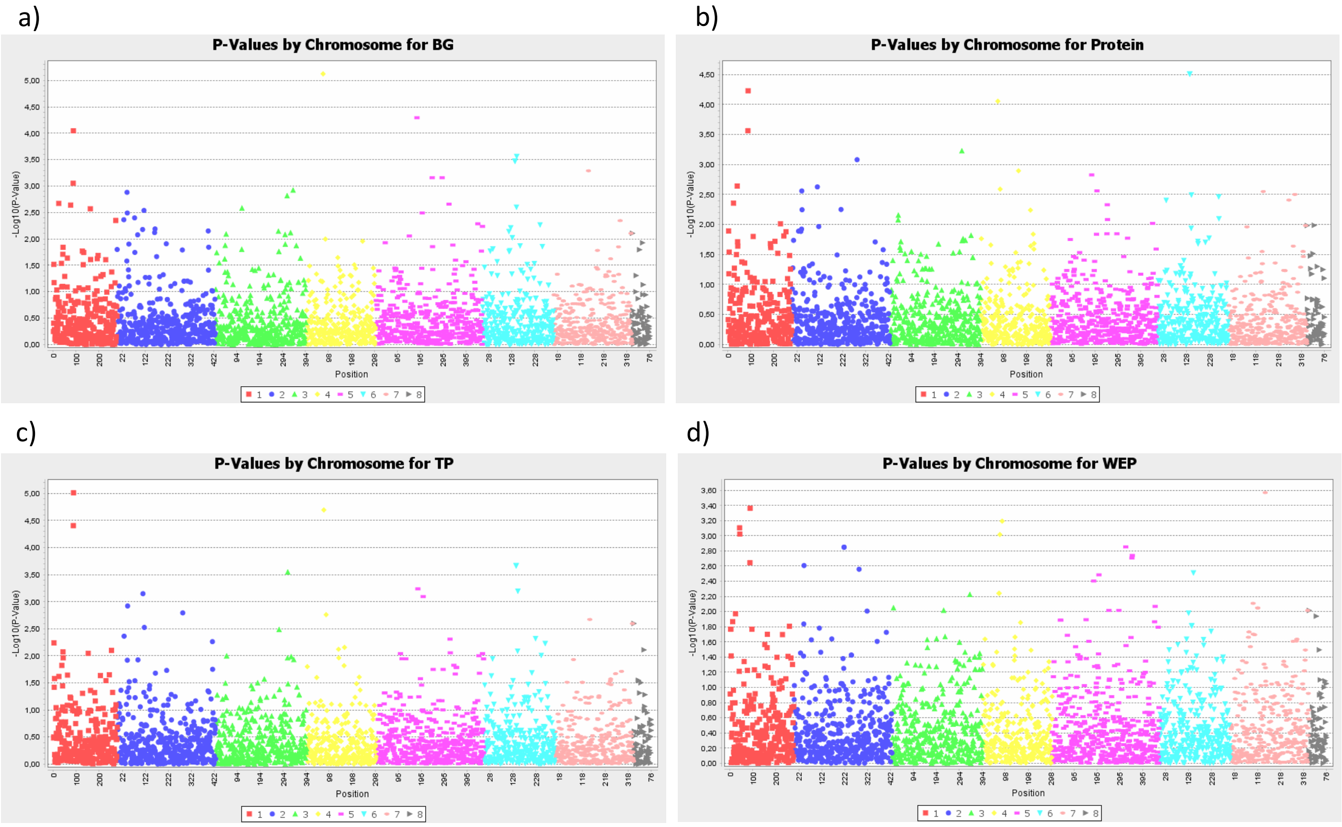


**Figure S5.** Manhattan plot represented the association of DArTSeq derived SNP markers with a) β-glucan (BG), b) protein, c) total-pentosan (TP) and d) water-extractable pentosan (WEP) traits in *Ae. biuncialis* population from mixed linear model (MLM).
